# Topical Medicine Potency of *Musa paradisiaca* var. *sapientum* (L.) kuntze as Oral Gel for Wound Healing: An *In Vitro* , *In Vivo* Study

**DOI:** 10.1055/s-0041-1740226

**Published:** 2022-02-18

**Authors:** Hendrik Setia Budi, Silvia Anitasari, Ninik Mas Ulfa, Wisnu Setyari Juliastuti, Mohammed Aljunaid, Doaa Elsayed Ramadan, Koko Muzari, Yung-Kang Shen

**Affiliations:** 1 Department of Oral Biology, Faculty of Dental Medicine, Universitas Airlangga, Surabaya, Indonesia; 2 Department of Dental Material and Devices, Faculty of Medicine, Universitas Mulawarman, Samarinda, Indonesia; 3 Department of Medical Microbiology, Faculty of Medicine, Universitas Mulawarman, Samarinda, Indonesia; 4 Surabaya Pharmacy Academy, Surabaya, Indonesia; 5 Faculty of Dental Medicine, Alsaeed University, Taiz, Yemen; 6 Ministry of Health and Population, Cairo, Egypt; 7 Faculty of Dental Medicine, Universitas Airlangga, Surabaya, Indonesia; 8 School of Dental Technology, College of Oral Medicine, Taipei Medical University, Taipei, Taiwan

**Keywords:** drug compounding, drug stability, health risk, oral gel, wound healing

## Abstract

**Objective** Topical application of ambonese banana ( *Musa paradisiaca* var. *sapientum* (L.) kuntze) stem sap gel (GEGPA) on the socket wound area showed an increase in the expression of platelet-derived growth factor-BB, while decrease in the expression of matrix metalloproteinase-2 and 9. The aim of this study is to achieve standard formulation of GEGPA through stability, viscosity, distribution area, and drugs release for oral gel wound healing.

**Materials and Methods** This is an *in vitro* and *in vivo* study with the randomized posttest only control group design. The gel was formulated according to the composition of each group by adding hydroxypropyl methylcellulose (HPMC), Lexgard, propylene glycol, and cold water to obtain 100 g of gel. Observations were made through the following tests: stability, viscosity, distribution area, drug release, and histopathological analysis of tooth extraction wound healing.

**Statistical analysis** Data were analyzed using a one-way analysis of variance ( *α* = 0.05) with GraphPad Prism-8 statistical software.

**Results** The study showed that the GEGPA formulation was stable against changes in consistency, color, smell, homogeneity, and pH value. There is a significant difference between groups with respect to viscosity ( *p* = 0.0001), adhesion ( *p* = 0.004), dispersion ( *p* = 0.000), and fibroblast cell numbers on days 3 and 5 ( *p* = 0.007 and *p* = 0.001). There is no interaction between the active ingredients and the gel base of all formulations. Formulation 3 had better properties in terms of viscosity, broad distribution, and drug release compared with other groups. Application of GEGPA to tooth extraction wounds showed a significant proliferation of fibroblast cells on days 3 and 5.

**Conclusions** The formulation of *M. paradisiaca* var. *sapientum* (L.) kuntze extract with HPMC and propylene glycol obtained a gel preparation, GEGPA, that was organoleptically stable and met the topical gel standard for wounds in the oral cavity.

## Introduction

Wound healing is a defense mechanism that works by repairing damaged cells or tissues. 1 2 The healing consists of a complex series of biological processes that involve cells and surrounding tissues supported by inflammatory and anti-inflammatory cytokines. The oral cavity is a remarkable environment in which wound healing process occurs in the presence of millions of microorganisms in warm oral fluids. 3 Topical or oral medications are used to localize wounds, relieve pain, prevent contamination, and promote healing. 4 5

The drug can be administered topically by applying it on the skin or mucosal surface. 6 Drugs that are commonly used for topical administration to the skin are usually in the form of creams, lotions, or ointments, 7 while drugs that are usually used for treating oral mucosa wounds are generally in the form of gels. 8 9 The success of topical treatment depends on patient age, selection of appropriate topical agents, location and area of the affected or diseased body, stage of disease, the concentration of active ingredients in the vehicle, methods of application, determination of drug-use duration, and penetration of the topical drug into the skin/mucosa. 10 11 12 Topical drug administration may be considered in patients with gastrointestinal disturbances, contraindications, or difficulty of swallowing. 13

Ambonese banana ( *Musa paradisiaca* var. *sapientum* (L.) kuntze) is one of the tropical plants that are often used as herbal medicines. The banana tree can be used as an alternative in the treatment of wounds, fevers, insect bites, digestive disorders, and epilepsy. 14 The stem sap extract of ambonese banana contains lectins, palmitic acid, leucocyanidin, quercetin, 3- *O* -galactoside, 3- *O* -glucoside, and 3- *O* -rhamnosyl glucoside, 15 and has analgesic, anti-inflammatory, antibacterial, 16 and antioxidant properties. 17 These also increase fibroblasts proliferation through the induction of growth factors such as *platelet-derived growth factor-BB* (PDGF-BB) and *transforming growth factor* - *β1* (TGF-β1). 18 The use of ambonese banana stem sap extract gel (GEGPA) in fibroblast cell culture shows that it is relatively nontoxic up to a concentration of 100%. 19 The increase in collagen in the wound area that was given the GEGPA can be caused by decrease in *matrix metalloproteinase-2* and *9* expressions, as well as the antioxidant activity of active compounds in it. 20 The gel consistency greatly affects the ease of its application on the oral wound area. The use of gel-forming materials such as hydroxypropyl methylcellulose (HPMC) in the preparation of ambonese banana stem sap extract has not been proven to interfere the wound healing process after extractions.

The gel is a semi-solid form that is clear, translucent, and contains active substances. It is also a colloidal dispersion that has force caused by tissues that are bonded to each other in the dispersed phase. 21 This gel form has several advantages such as its nonstickiness, the little concentration of gelling agents that is required to form good gel mass, and the gel viscosity that will not change significantly at storage temperature. 22 Every drug should have a good stability; therefore, the pharmaceutical form must be considered carefully. This is very important considering the drug forms that are generally made in large quantities and require long-term storage. If a drug is stored for a long period of time, it can decompose and result in the reduced effects received by the patient. 23 A good formulation is needed for optimum drug stability and the factors that influence the stability of a new drug. This study aims to obtain a suitable gel formulation, so that the stability, viscosity, distribution, and release of active compounds in ambonese banana stem sap extract are maintained as a potential drug.

## Materials and Methods

### Ambonese Banana Stem Sap Preparation

The plant was determinated in the Lembaga Ilmu Pengetahuan Indonesia, Purwodadi, East Java. Ambonese banana stems were cleaned of dirt with running water. The sap from the ambonese banana stem was taken by extracting it. The stems were cut into thin strips with a thickness of 0.5 to 1 cm and dried using an oven (MMM, GmbH) at 40°C for 3 hours. The dried stems were made into powder and then weighed as much as 1 kg. The maceration process was performed with 2 liters of 96% ethanol. The maceration process with ethanol solvent was performed twice, up to 7 days. The results of the maceration were filtered using filter paper (Whatman number 41), and then separated between the extract and solvent using an evaporator (Heidolph-VAP, Germany) at 40°C with 200 rpm speed for 2 hours, to obtain a thick extract. The extract was stored in a closed, dark container and protected from light.

### Ambonese Banana Stem Sap Gel (GEGPA) Formulation

The gel was formulated according to the composition in Table 1 , and the required ingredients were weighed. HPMC was dispersed into 30 mL of water at 80 to 90°C until it expanded. It was then stirred until a clay (gel) was formed. The stirring process must be in a cold state such as a basin that had been given ice cubes. Lexgard was mixed in propylene glycol according to the groups (F1, F2, F3); then the extract was added until it was properly mixed. The mixture was added to the HPMC mixture and stirred until it was homogeneous. Cold water was added to get 100 g of gel; then it was packed in a tightly sealed tube. In the control group (K), no extract was added.

**Table 1:** The formulation of the ambonese banana hump extract gel (GEGPA)

Material	Material concentration (% b/b)		
	F1	F2	F3
Active material	1	1	1
HPMC	4	4	4
Propylene glycol	10	15	20
Lexgard	0.01	0.01	0.01
Aqua ad	100	100	100

Abbreviations: F1, formulation 1; F2, formulation 2; F3, formulation 3; HPMC, hydroxypropyl methylcellulose.

### Gel Stability Test

The stability of GEGPA was tested at room temperature by observing (organoleptic) the color, smell, homogeneity, pH, and viscosity. 24 Samples were stored at 4°C for 24 hours. After that, the samples were taken out and placed at 40°C for 24 hours. This treatment is counted as one cycle. The experiment was repeated for six cycles while taking the physical conditions into account during the experiment by comparing the previous forms.

### Gel pH Test

pH testing on GEGPA was done by inserting the pH meter electrode (Mettler Toledo S220, Merck) into the gel. 24 The gel's pH value will be displayed on the pH meter's screen. The recording of the pH value is awaited until the number on the screen does not change (stable). A suitable gel preparation has a pH value that is near to the oral pH, which is between 6.2 and 7.6.

### Gel Viscosity Test

The viscosity test is used to determine the viscosity of the gel preparation by using a viscometer (Haake 6R viscometer, Thermo Scientific, Germany). 24 The gel preparation sample was put into a glass beaker and placed under a spindle hanger. The spindle is installed on the spindle hanger; then it was lowered to the limit immersed in the extract gel preparation. Next, the rotor was turned on while pressing the button. The spindle was allowed to rotate and the red needle was observed on the scale; then the number indicated by the needle was read.

### Gel Homogeneity Test

The homogeneity test was performed by weighing 0.1 g of each sample. Furthermore, the sample is smeared on an object glass, and the homogeneity of the preparation and the presence of air bubbles were observed under a microscope at a 100× magnification. 24

### Gel Adhesion Test

The adhesion test was performed by means of 0.25 g of sample placed on top of two predetermined glass objects, then pressed with a load of 1 kg for 5 minutes. The glass object was put on the test equipment and then a load of 80 g was added. The time it takes for the glass object to come off is the stickiness ability of the gel that is going to be recorded and tabulated. 25

### Gel Dispersion Test

The dispersion test was performed by weighing the gel preparation as much as 0.5 g; after that the gel was placed right under a round glass underneath, accompanied by a diameter scale. Next, it was covered with another glass that had been weighed and then allowed to sit for 1 minute. The scatter diameter of each gel was then measured. After 1 minute, a 50 g load was added and allowed to sit for 1 minute. Next, the diameter of the spread was remeasured. This was repeated every 1 minute with the addition of a 50 g load until a sufficient diameter was obtained to observe the effect of the load on the diameter of the spread of the gel preparation. 25

### Power of Diffusion and Permeation Tests

The gel diffusion test was performed with a Millipore synthetic membrane, which was placed between two halves of the diffusion cell. Then, the receptor was filled with phosphate buffer of 6.8 pH and 5% ethanol to 130 mL at 37°C. One gram of gel was applied to the membrane. The absorption was measured every 30 minutes to 8 hours at a 274 nm wavelength with an ultraviolet-visible spectrophotometer (Shimadzu 1800). 25

### Histopathology of Fibroblast Cells on Tooth Socket Wound Healing

This research has passed the health ethics test with number: 586/HRECC.FODM/IX/2020. The experimental animals were 24 male rats, Wistar type, 2 to 3 months old, and divided into 4 groups. Rats were anesthetized according to their body weight (30 mg/kg thionembutal). After the rats were anesthetized, the wound was made by extracting the mandibular left incisor. 26 In the control group (K), the socket was only given the drug carrier gel. The treatment group was given GEGPA with different formulations, namely F1, F2, and F3. The gel was injected into the dental socket of Wistar rats using modified needle (as much as 1 to 2 mL of tuberculin syringe). After tooth extraction, the wound was closed by suturing using 5–0 Vicryl (Ethicon, Johnson & Johnson do Brasil, São Jose dos Campos, SP, Brazil). Decapitation was performed on days 3 and 5; then the tissue was washed to remove the residual blood with 0.9% sodium chloride physiological solution and fixed using 10% neutral buffer formalin. The mandible bone was decalcified using 10% ethylenediaminetetraacetic acid (Sigma) and then paraffin blocks were made. The preparations were made with a 4 µ thickness for histological examination of fibroblast cells in the apical one-third of the extraction socket. The preparations were observed using a light microscope (Nikon H600L) with 400× magnification in five fields of view, equipped with a calibrated micrometer. The preparations were processed using the Nikon Image System software and a 300 megapixels DS Fi2 digital camera.

### Statistical Analysis

Data were tabulated and analyzed using a one-way analysis of variance (ANOVA) test with *GraphPad Prism-8* statistical software at 95% significance. Differences in the mean value of viscosity, adhesion, dispersion, and the number of fibroblasts were analyzed statistically by one-way ANOVA. The gel diffusion and permeation data were analyzed by regression.

## Results

### Organoleptic Observations of GEGPA

The four formulations showed that in the control group a clear, homogeneous gel was formed, with a typical smell of gel base, 6.8 pH, and thick viscosity. In the three groups of GEGPA formulations, there are no organoleptic differences between the gel samples. The gel is clear brown, homogeneous, has a typical banana smell, and thick viscosity. The experiment was repeated for six cycles and compared with the previous forms. Physically, no significant change is found between groups with respect to color, smell, homogeneity, pH, and viscosity, but there is a decrease in the pH of 0.1 in the F1 and F2 groups. The formulation of F3 is more stable compared with the treatment groups ( Table 2 ).

**Table 2:** Organoleptic observations of gel formulations

Physical properties test	Formula			
	Control (Base)	F1	F2	F3
Color	Clear	Clear brown	Clear brown	Clear brown
smell	Typical base	Specialty banana	Specialty banana	Specialty banana
pH	6.8	6.7	6.7	6.8
Viscosity	Thick	Thick	Thick	Thick
Homogeneity	Homogeneous	Homogeneous	Homogeneous	Homogeneous

Abbreviations: F1, formulation 1; F2, formulation 2; F3, formulation 3; HPMC, hydroxypropyl methylcellulose.

Note: Description: Control, base (HPMC 4%), F1, Eq. 1, F2; Eq. 2; F3, Eq. 3.

### The Difference in Gel Viscosity of GEGPA

**Fig. 1:**
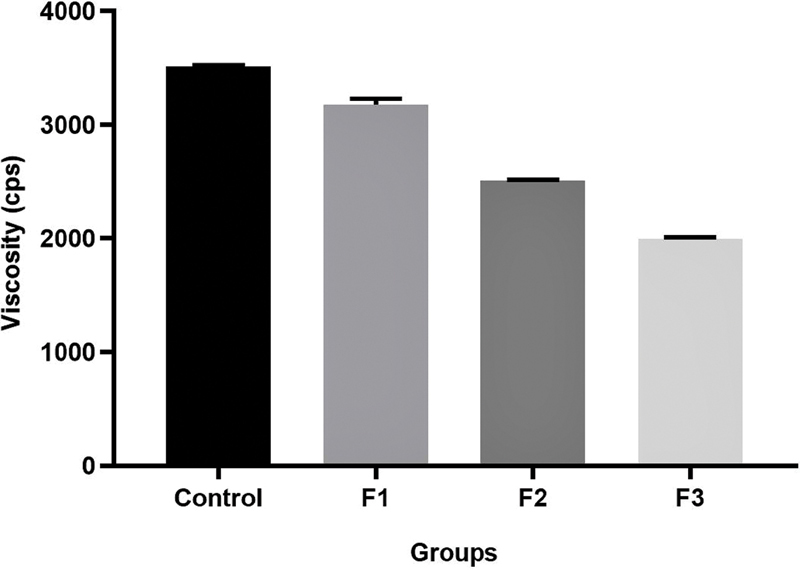
Viscosity of ambonese banana stem sap extract gel. F1, formulation 1; F2, formulation 2; F3, formulation 3.

Viscosity testing aims to determine the value of the viscosity of a substance. The higher viscosity of the gel will make the gel thicker resulting in more difficult drug to spread. There is a decrease in the viscosity of each formulation with increase in the volume of propylene glycol that had been used. Sequentially, the highest viscosity values are: the control group > F1 > F2 > F3, with a value of 3,592; 3,187; 2,438; and 2,035 cps ( Fig. 1 ). There is a significant difference in the viscosity values of each group ( *p* = 0.0001).

### Gel Adhesion Ability of GEGPA

**Fig. 2:**
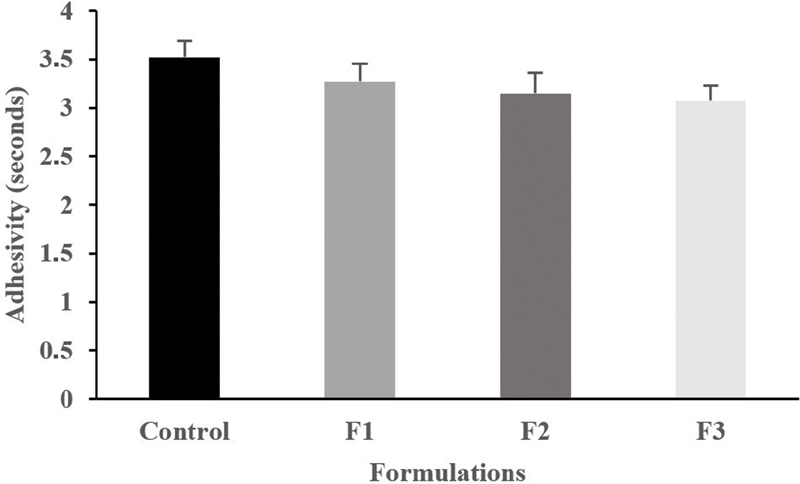
Average gel adhesion strength. F1, formulation 1; F2, formulation 2; F3, formulation 3.

The gel adhesion test is intended to determine the ability of the gel to adhere to the oral mucosa. The gel adhesion test was evaluated by observing the length of time the glass objects remained attached. There is a significant decrease in the length of attachment time in each group ( *p* = 0.004). Obviously, the GEGPA formulation can affect the time length of adhesion ( Fig. 2 ).

### Gel Dispersion of GEGPA

**Fig. 3:**
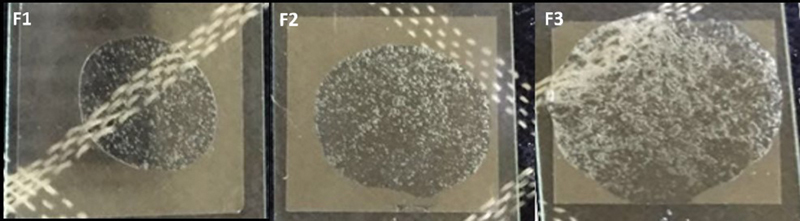
Differences in the spread of gel in ambonese banana stem sap gel groups. F1, formulation 1; F2, formulation 2; F3, formulation 3.

**Fig. 4:**
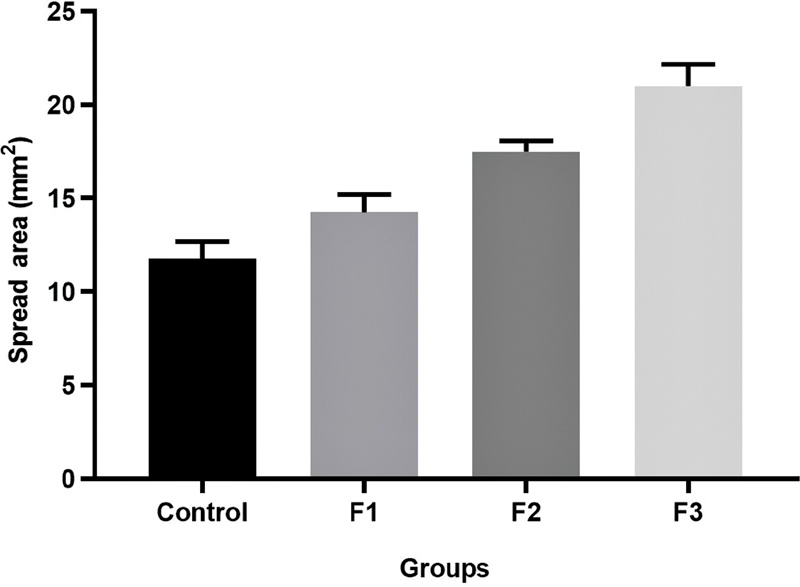
Differences in dispersion between groups of gels. F1, formulation 1; F2, formulation 2; F3, formulation 3.

Gel examination is intended to determine the ability of the gel to spread in the oral mucosa. The GEGPA distribution in the F3 group was wider than in the other groups ( Fig. 3 ). Sequentially, the widest dispersion ability is: F3 > F2 > F1 > control, with a value of 21.76, 17.91, 14.05, and 12.37 mm ^2^ ( Fig. 4 ). There is a significant difference in dispersion ability between groups ( *p* = 0.000).

### Diffusion and Penetration of GEGPA across Membranes

**Fig. 5:**
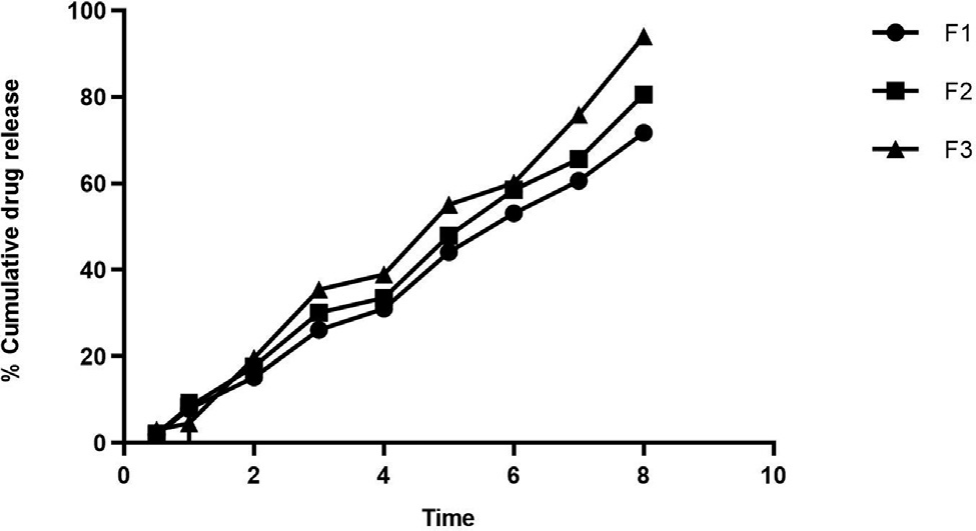
Diffusion and permeation power of ambonese banana stem sap gel. F1, formulation 1; F2, formulation 2; F3, formulation 3.

As per the results ( Fig. 5 ), the spectrophotometer data for the maximum absorbance of the gel are at 274 nm wavelength in a 6.8 phosphate buffer solution. Referring to Lambert Beer's law of the standard solution with a concentration of 10 to 180 µg, it is found that the linearity ( *r* ^2^ ) is 0.999, and regression value ( *Y* ) is 0.0171X + 0.003. From this graph, the results indicate that there is no interaction between the active ingredients and the gel base of all formulations (F1, F2, and F3).

### Fibroblast Proliferation in the Healing Process of Tooth Sockets

**Fig. 6:**
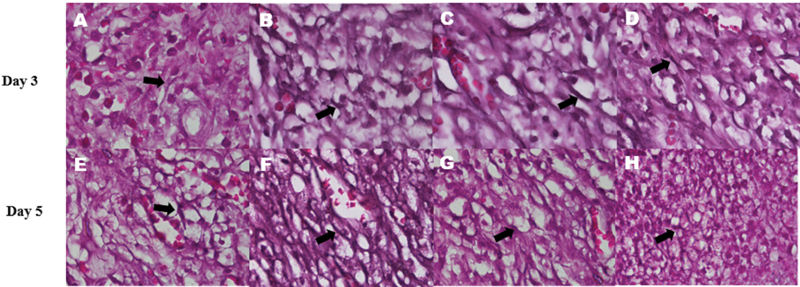
The difference in the number of fibroblast cells (arrows) between groups with hematoxylin-eosin staining. Fibroblast cells proliferation on day 3: Control ( **A** ), formulation F1 ( **B** ), formulation F2 ( **C** ), and formulation F3 (D). Fibroblast cells proliferation on day 5: Control ( **E** ), formulation F1 ( **F** ), formulation F2 ( **G** ), and formulation F3 ( **H** ). 400x magnification, Nikon H600L microscope, DS Fi2 camera 300 megapixels.

There is a significant increase in the number of fibroblast cells in the tooth socket on healing days 3 and 5 in the groups that were given GEGPA, which are F1, F2, and F3, compared with the control group ( *p* = 0.007 and *p* = 0.001; Table 3 ). The apical one-third of the socket in groups F1, F2, and F3 appears to be filled with fibroblast cells more rapidly ( Fig. 6 ). There is no significant difference in the number of fibroblasts in the F1, F2, and F3 groups on days 3 and 5 ( *p* = 0.207 and *p* = 0.112). The number of fibroblast cells in the control group appears to be lesser than the group given GEGPA.

**Table 3:** The average number of fibroblast cell proliferation on days 3 and 5

Groups	Count of fibroblast cells (x̄ ± SD)			
	Day 3	*p-* Value	Day 5	*p-* Value
Control	8.3 ± 0.52	0.007 **a**	12.1 ± 0.86	0.001 **a**
F1	16.7 ± 0.75		22.3 ± 1.13	
F2	18.1 ± 0.66		24.3 ± 1.02	
F3	8.3 ± 0.52		25.7 ± 1.15	

Abbreviations: SD, standard deviation; x̄, mean.

a *p-* Value, significance level 0.05.

## Discussion

A lot of research has been done to get a material that can accelerate the wound healing process in the oral cavity, as well as reduce postoperative complications. In controlling complex microorganisms in the oral cavity, topical wound protection agents are surely needed, besides oral drugs to reduce pain, inflammation, and infection. Oral drug use will give absorption, metabolism, distribution, and excretion (ADME) effect, as well as first-pass metabolism. 27 28 Disruption of ADME results in reduced drug availability in the systemic circulation, and low-found drug concentrations in tissue. 29 Recent researches in dentistry are mainly focused on the development of local agent's use or drugs that are applied directly to the wound area. It is hoped that the local/topical administration will be more effective because it can be absorbed directly and interact with surrounding cells and tissues. Oral gel formulations may reduce side-effects in patients with gastrointestinal disorders if taken orally, or in patients with medical conditions such as dysphagia. 30 In addition, there are many oral gel products that are available commercially, most of which are quick-release formulations that release the active ingredient by diffusion. 31

In this study, HPMC was used for making the GEGPA preparations, which act as a gelling agent. HPMC will form a colloidal system when dissolved in water. Lipophilic colloids are generally organic molecules that can be dissolved with molecules from the dispersing phase. 22 HPMC is nontoxic and has good water binding ability. 32 The use of 4% HPMC in GEGPA preparations produces the appropriate viscosity and can hold blood out of wounds as found in the result of the extraction of rat teeth. 18 20 A topical preparation's solubility can be increased by adding a cosolvent or a solubility-enhancing compound. One of the cosolvents that is often used in gel preparations is propylene glycol with a concentration of 5 to 80%. 33

A formulation of propylene glycol cosolvent with a concentration of 10 to 20% was used for oral gel preparations. The use of propylene glycol can also increase drug penetration. 34 Propylene glycol is used because it has hydrophilic and hydrophobic groups, so it can dissolve hydrophobic compounds. 35 In addition, propylene glycol is a cosolvent with low toxicity. 36 GEGPA formulations that consist of ambonese banana stem extracts, HPMC, and propylene glycol, produced a brownish clear gel with thick viscosity and a typical banana scent. The GEGPA formulation has a slightly decreased pH, namely by 0.1, compared with the pH of the control group, which is 6.8. The pH value of GEGPA still meets the requirements for a normal oral pH environment, which is between 6.7 and 7.3, and saliva with a pH between 6.2 and 6.7. 37 The use of 20% propylene glycol can balance the pH of GEGPA, so that it becomes the same as the pH of the control group. It is possible because in GEGPA preparations, the compound in ambonese banana stem sap can lower the pH of propylene from 9.0–10.5 to 6.7–6.8.

The viscosity of the GEGPA formulation is also less compared with the control group. A good gel has a viscosity between 2,000 and 4,000 cps. 38 39 The GEGPA formulation has a viscosity between 2,035 and 3,187 cps, so the results of this study indicate that the GEGPA formulation meets the requirements of a fairly good oral gel. Due to the encouragement of blood flow that comes out of the wound, the appropriate gel viscosity can inhibit gel detachment from the mucosa.

The thicker the gel or the higher the viscosity of the gel, the more difficult it is for the active ingredient to be separated from the carrier material and the longer is the penetration time into the tissue, so the absorption of the active ingredient can decrease. The increase in viscosity can also affect the adhesion of the gel to the mucosa. The gel is considered appropriate when the drug can diffuse quickly into the wound.

The dispersion of the gel is increased at a low viscosity. Viscosity affects the dispersed area. Thus, the greater the viscosity value of the gel preparation, the greater is the resistance of the gel preparation to spread, resulting in a decreasing dispersion value. Conversely, if the viscosity value of the gel preparation is getting smaller, the resistance of the gel preparation to spread will also be getting smaller, and so it will have a wide dispersion. The spreadability of the GEGPA formulation in the F3 group is wider than in the other groups. So, when applied to the application site, this ensures that the formulation maintains a good wet contact time.

Therefore, the active ingredients used in these formulations can be combined with the excipients used in all of these gel formulations. From the comparison of the three formulations above, it can be seen that the third formulation experienced the best release of active ingredients compared with other formulations. In an *in vitro* study, it was found that formula 3 was released by permease diffusion of 94.01% for 8 hours, so that formula 3 can describe the increase of the active ingredient penetration and absorption.

The patient's well-being may be harmed by a delayed tooth extraction socket healing process, which increases the risk of infection. The proliferation phase of fibroblast proliferation is a crucial stage in the healing process. Bone development in the socket is facilitated by fibroblasts made up of extracellular matrix and collagen fibers. Fibroblasts are particularly critical cells in the early stages of wound healing, and they begin to form on the third day following injury during the proliferative stage. Fibroblasts begin to move to the wound site and proliferate between days 3 and 5, resulting in a dominance of their numbers on the wound site. 40 The use of GEGPA inserted into the tooth sockets of mice shows an increase in the number of fibroblasts on days 3 and 5. Phytochemical compounds in GEGPA such as lectins, palmitic acid, leucocyanidin, quercetin, 3-O-galactoside, 3-O-glucoside, and 3-O-rhamnosyl glucoside can be released from the gel, and play an important role in activating PDGF-BB and TGF-β1, 18 so that they can increase fibroblasts' proliferation in the tooth socket area. The distribution of these compounds in the form of a gel can certainly accelerate wound healing.

## Conclusions

The formulation of *M. paradisiaca* var. *sapientum* (L.) kuntze extract with HPMC and propylene glycol obtained a gel preparation, GEGPA, that was organoleptically stable and met the topical gel standard for wounds in the oral cavity.

## References

[1] Rodrigues M Kosaric N Bonham C A Gurtner G C Wound healing: a cellular perspective Physiol Rev 2019 99 01 665 706 30475656 10.1152/physrev.00067.2017 PMC6442927

[2] Gonzalez A C Costa T F Andrade Z A Medrado A R Wound healing - a literature review An Bras Dermatol 2016 91 05 614 620 27828635 10.1590/abd1806-4841.20164741 PMC5087220

[3] Cho Y D Kim K H Lee Y M Ku Y Seol Y J Periodontal wound healing and tissue regeneration: a narrative review Pharmaceuticals (Basel) 2021 14 05 456 10.3390/ph14050456 34065862 PMC8151433

[4] Abbas Shamash M S Zaidan T F Curcumin modulate TGFßii-R to improve healing of oral ulceration Int J Pharm Res 2020 12 1288 1294

[5] Sood A Granick M S Tomaselli N L Wound dressings and comparative effectiveness data Adv Wound Care (New Rochelle) 2014 3 08 511 529 25126472 10.1089/wound.2012.0401 PMC4121107

[6] Leppert W Malec-Milewska M Zajaczkowska R Wordliczek J Transdermal and topical drug administration in the treatment of pain Molecules 2018 23 03 681 29562618 10.3390/molecules23030681 PMC6017304

[7] Garg T Rath G Goyal A K Comprehensive review on additives of topical dosage forms for drug delivery Drug Deliv 2015 22 08 969 987 24456019 10.3109/10717544.2013.879355

[8] Ibrahim S A Elkot R A Soliman H E Lactic acid 5% mouth wash vs Kenalog in Orabase 0.1% for treatment and prophylaxis of recurrent aphthous ulcer J Cosmet Dermatol 2020 19 04 964 969 31436378 10.1111/jocd.13110

[9] Hammad H M Hammad M M Abdelhadi I N Khalifeh M S Effects of topically applied agents on intra-oral wound healing in a rat model: a clinical and histomorphometric study Int J Dent Hyg 2011 9 01 9 16 21226845 10.1111/j.1601-5037.2009.00410.x

[10] Rajabalaya R Musa M N Kifli N David S R Oral and transdermal drug delivery systems: role of lipid-based lyotropic liquid crystals Drug Des Devel Ther 2017 11 393 406 10.2147/DDDT.S103505 PMC5315216 28243062

[11] Ruela A LM Perissinato A G Lino M de S Mudrik P S Pereira G R Evaluation of skin absorption of drugs from topical and transdermal formulations Braz J Pharm Sci 2016 52 03 527 544

[12] Benson H AE Grice J E Mohammed Y Namjoshi S Roberts M S Topical and transdermal drug delivery: from simple potions to smart technologies Curr Drug Deliv 2019 16 05 444 460 30714524 10.2174/1567201816666190201143457 PMC6637104

[13] Kappelle W FW Siersema P D Bogte A Vleggaar F P Challenges in oral drug delivery in patients with esophageal dysphagia Expert Opin Drug Deliv 2016 13 05 645 658 26781167 10.1517/17425247.2016.1142971

[14] Kumar K PS Bhowmik D Duraivel S Umadevi M Traditional and medicinal uses of banana J Pharmacogn Phytochem 2012 1 03 51 63

[15] Imam M Z Akter S Musa paradisiaca l. and musa sapientum l.: a phytochemical and pharmacological review J Appl Pharm Sci 2011 1 05 14 20

[16] Budi H S Juliastuti W S Christy B R Antimicrobial activity of musa paradisiaca var. sapientum on enterococcus faecalis viability Malays J Med Health Sci 2020 16 04 17 21

[17] Abdel Ghany T M Ganash M Alawlaqi M M Al-Rajhi A MH Antioxidant, antitumor, antimicrobial activities evaluation of musa paradisiaca l. Pseudostem exudate cultivated in Saudi Arabia Bionanoscience 2019 9 01 172 178

[18] Budi H S Soesilowati P Imanina Z Gambaran histopatologi penyembuhkan luka pencabutan gigi pada makrofag dan neovaskular dengan pemberian getah batang pisang ambon Maj Kedokt Gigi Indones 2017 3 03 3 9

[19] Budi H S Juliastuti W S Ariani W Mtt-based cytotoxic evaluation of ambonese banana stem sap (Musa paradisiaca var. Sapientum (L.) Kuntze) on FIBROBLAST CELLS Periód Tchê Quím 2020 17 36 624 633

[20] Budi H S Astuti E R The MMP-2, MMP-9 expression and collagen density of the ambonese banana stem sap administration on wound healing J Int Dent Med Res 2019 12 02 492 497

[21] Allen L v Ansel H C Ansel' s Pharmaceutical Dosage Forms and Drug Delivery Systems. Ansel' s Pharmaceutical Dosage Forms and Drug Delivery Systems 10th edition. Philadelphia Lippincott Williams & Wilkins 2014

[22] Noval N Rosyifa R Annisa A Effect of HPMC concentration variation as gelling agent on physical stability of formulation gel ethanol extract bundung plants (Actinuscirpus grossus)Proceedings of the First National Seminar Universitas Sari Mulia, NS-UNISM 2019, 23rd November 2019, Banjarmasin, South Kalimantan, Indonesia 10.4108/eai.23-11-2019.2298326

[23] Gikonyo D Gikonyo A Luvayo D Ponoth P Drug expiry debate: the myth and the reality Afr Health Sci 2019 19 03 2737 2739 32127846 10.4314/ahs.v19i3.49 PMC7040264

[24] Patil A Bhide S Bookwala M Stability of organoleptic agents in pharmaceuticals and cosmetics AAPS PharmSciTech 2018 19 01 36 47 28900868 10.1208/s12249-017-0866-2

[25] Ueda C T Shah V P Derdzinski K Topical and transdermal drug products Pharmacop Forum 2010 17 04 750 764

[26] Khoswanto C A new technique for research on wound healing through extraction of mandibular lower incisors in Wistar rats Eur J Dent 2019 13 02 235 237 31466118 10.1055/s-0039-1694312 PMC6777148

[27] Raj G M Raveendran R Introduction to Basics of Pharmacology and Toxicology: Volume 1: General and Molecular Pharmacology: Principles of Drug Action 1st edition. Singapore Springer 2019

[28] Chillistone S Hardman J G Modes of drug elimination and bioactive metabolites Anaesth Intensive Care Med 2020 21 09 388 391

[29] Caldwell J Gardner I Swales N An introduction to drug disposition: the basic principles of absorption, distribution, metabolism, and excretion Toxicol Pathol 1995 23 02 102 114 7569663 10.1177/019262339502300202

[30] Drumond N Stegemann S Better medicines for older patients: considerations between patient characteristics and solid oral dosage form designs to improve swallowing experience Pharmaceutics 2020 13 01 32 10.3390/pharmaceutics13010032 33379258 PMC7824227

[31] Hishikawa Y Kakino Y Tsukamoto H Tahara K Onodera R Takeuchi H Control of drug diffusion behavior of xanthan and locust bean gum gel by agar gel Chem Pharm Bull (Tokyo) 2016 64 10 1450 1457 27725500 10.1248/cpb.c16-00135

[32] Popov T A Emberlin J Josling P Seifalian A In vitro and in vivo evaluation of the efficacy and safety of powder hydroxypropylmethylcellulose as nasal mucosal barrier Med Devices (Auckl) 2020 13 107 113 32308507 10.2147/MDER.S236104 PMC7136663

[33] Rowe R C Paul J S Owen S C Handbook of Pharmaceutical Excipients London Pharmaceutical Press 2006

[34] Allen L V BLT in propylene glycol topical gel US Pharm 2013 38 12 36 37

[35] Nayak A K Panigrahi P P Solubility enhancement of etoricoxib by cosolvency approach ISRN Phys Chem 2012 2012 820653 10.5402/2012/820653

[36] Jagtap S Magdum C Jadge D Jagtap R Solubility enhancement technique: a review J Pharm Sci Res 2018 10 09 2205 2211

[37] Baliga S Muglikar S Kale R Salivary pH: a diagnostic biomarker J Indian Soc Periodontol 2013 17 04 461 465 24174725 10.4103/0972-124X.118317 PMC3800408

[38] Garg A Aggarwal D Garg S Singla A K Spreading of semisolid formulations: an update Pharm Technol 2002 26 09 84 105

[39] Nurman S Yulia R Noor E Sunarti T C The optimization of gel preparations using the active compounds of arabica coffee ground nanoparticles Sci Pharm 2019 87 04 32

[40] Amaliya A Muhaimina R K Susanto A Sutjiatmo A B Histological assessment of palatal donor site wound healing after application of Moringa oleifera Lamarck leaf extract in rats Eur J Dent 2019 13 02 248 254 31509874 10.1055/s-0039-1695065 PMC6777152

